# Epitranscriptomics in poultry: RNA modifications as emerging regulators of growth, immunity, and metabolism: a narrative review

**DOI:** 10.3389/fvets.2026.1950849

**Published:** 2026-10-26

**Authors:** Muhammad Iftikhar, Mohamed Tharwat, Rifat Ullah Jan, Aftab Shaukat, Nourhan Nassar, Asfand Yar Khan, Fahad A. Alshanbari, Shahab Ur Rehman

**Affiliations:** 1 College of Animal Sciences and Technology, Yangzhou University, Yangzhou, Jiangsu, China; 2 Department of Animal Medicine, Faculty of Veterinary Medicine, Zagazig University, Zagazig, Egypt; 3 Animal Science Division, Department of Modern Agriculture, College of Life and Environmental Sciences, Hunan University of Arts and Science, Changde, Hunan, China; 4 College of Veterinary Medicine, Huazhong Agricultural University, Wuhan, China; 5 Department of Clinical Sciences, Faculty of Veterinary Sciences, The University of Veterinary and Animal Sciences, Swat, Pakistan; 6 Department of Medical Biosciences, College of Veterinary Medicine, Qassim University, Buraidah, Saudi Arabia

**Keywords:** epitranscriptomics, immunity, N6-methyladenosine, nanopore direct RNA sequencing, poultry, RNA modifications

## Abstract

Epitranscriptomics has recently emerged as a dynamic layer of gene expression regulation, in which chemical modifications in RNA molecules, such as N6-methyladenosine (m6A), 5-methylcytosine (m5C), N1-methyladenosine (m1A), pseudouridine (Ψ), and 2′-O-methylation, modulate RNA stability, splicing, translation efficiency, and cellular responses to environmental stimuli. Although researchers have characterized >170 distinct RNA modifications across a broad range of organisms, the biological significance of these epitranscriptomic marks in birds remains largely unknown. This knowledge gap is especially important given the global economic impact of poultry production and the need to understand the molecular mechanisms that regulate growth efficiency, metabolic adaptation, immune competence, and disease resistance in chickens and other poultry species. Recent technological advances, notably nanopore direct RNA sequencing, enable direct analysis of native RNA molecules while preserving modification-associated signal patterns and avoiding reverse-transcription and amplification biases. However, the accuracy and resolution of modification detection depend on the modification type, sequencing chemistry, analytical model, read coverage, and availability of appropriate controls, and therefore often require orthogonal validation. These advances, especially in combination with integrative multi-omics approaches, provide unprecedented opportunities to map tissue-specific and condition-dependent epitranscriptomic landscapes in poultry. Here, we summarize the current knowledge of RNA modification mechanisms, review the available technologies for their detection with a focus on their application in poultry research, and critically evaluate emerging evidence that RNA modifications regulate muscle development, nutrient sensing, metabolic homeostasis, and innate and adaptive immunity. We then review the translational potential of epitranscriptomics for precision poultry nutrition, marker-assisted breeding programs, and novel disease control strategies, and identify key knowledge gaps and future research priorities. This review aims to bridge the gap between molecular understanding and practical application and provides a framework for incorporating epitranscriptomics into poultry science and sustainable livestock production.

## 1 Introduction

Epitranscriptomics has recently emerged as a fundamental layer of gene regulation, complementing genomic, epigenomic, and transcriptomic control of cellular processes. Compared with DNA-level epigenetic changes, epitranscriptomic regulation involves adding chemical modifications directly to RNA molecules. These changes can influence RNA processing, stability, localization, and translation efficiency. To date, more than 170 RNA modifications have been identified across different RNA types, including messenger RNA (mRNA), ribosomal RNA (rRNA), transfer RNA (tRNA), and several types of non-coding RNAs (1, 2). N6-methyladenosine (m6A), the most common internal modification in eukaryotic mRNAs, helps regulate many aspects of RNA metabolism, including splicing, nuclear export, translation, and degradation. Most eukaryotes possess numerous additional types of RNA modifications including, but not limited to, 5-methylcytosine (m5C), N1-methyladenosine (m1A), Pseudouridine (Ψ), and 2’-O-methylation. Additions of this nature contribute to the overall complexity of RNA molecular network regulation. These RNA modifications are dynamically regulated by specialized molecular machines commonly called “writers” (add modifications), “readers” (recognize modifications), and “erasers” (remove modifications) (3, 4). These regulatory proteins work together to enable cells to rapidly and accurately alter gene expression programs in response to developmental cues, metabolic signals, and environmental stresses (5). These advanced enzymatic systems that mediate the deposition, removal, or recognition of epitranscriptomic marks are key post-transcriptional regulators that control cellular phenotypic plasticity (6). In avian species, such alterations are increasingly recognized as important coordinators of physiological traits ranging from developmental growth and reproductive efficiency to the modulation of immune responses against viral pathogens (7, 8).

Although direct functional evidence in poultry remains limited, this review interprets the evidence discussed at three levels based on three levels. First, direct poultry evidence refers to studies in chickens or other domesticated avian species where RNA modifications were experimentally profiled or functionally manipulated (3, 9). Second, comparative evidence refers to mechanistic findings from mammals or other non-avian model systems that may inform poultry biology because of conserved RNA-modification machinery (10). Third, the proposed poultry mechanisms are hypotheses that must be experimentally tested in avian systems. Associative findings from MeRIP-seq, differential methylation analysis, transcriptomic correlation or pathway-enrichment analysis are considered associative and not causal unless validated by functional perturbation, rescue experiments or independent mechanistic evidence (11).” This structured approach acknowledges the high evolutionary conservation of epitranscriptomic regulators, such as methyltransferases and demethylases, which facilitate the dynamic and reversible nature of these chemical signatures across vertebrate species (12, 13).

RNA modifications play an essential role in several biological processes in mammals, such as early embryonic development, immune response, metabolic homeostasis, and disease development (14). Despite growing awareness of epitranscriptomic regulation in vertebrate biology, much less information remains on RNA modifications in birds. In particular, chickens are one of the most important agricultural models for studying the regulation of metabolism, immune response, and growth ability (15). Research into epitranscriptomic mechanisms may provide new insight into molecular pathways related to productivity traits, resistance to infectious agents, and adaptation to different nutritional conditions. Recent advancements in high-throughput sequencing technologies have significantly expedited the study of epitranscriptomics compared to past years, while providing many new options for characterizing RNA modification landscapes across tissues/developmental time frames/environments (16). Previously, transcriptome-wide profiling of individual RNA modifications relied largely on antibody-based enrichment methods, in which modification-specific antibodies capture RNA fragments carrying a particular mark. For example, MeRIP-seq profiles N6-methyladenosine (m6A), m5C-RIP-seq profiles 5-methylcytosine (m5C), and miCLIP enables m6A and m6Am mapping at single-nucleotide resolution. Because each antibody recognizes only its cognate modification, these methods profile one modification type per experiment rather than the entire epitranscriptome, and they typically do not provide nucleotide-level resolution unless crosslinking-based approaches are used. The advent of third-generation sequencing techniques, most notably nanopore direct RNA sequencing, has opened new possibilities in this field. Nanopore direct RNA sequencing analyzes native RNA molecules without reverse transcription or amplification, and chemical modifications can be inferred from modification-associated changes in the electrical current signal as the RNA traverses the pore. However, the accuracy and resolution of such calls depend on the modification type, sequencing chemistry, analytical model, read coverage, and the availability of appropriate control datasets, and site-level assignments therefore often require orthogonal validation (17–19).

In this review, we provide a comprehensive overview of epitranscriptomic regulation and discuss emerging evidence suggesting that RNA modifications may play important roles in poultry growth, metabolic regulation, immune responses, and disease susceptibility. We also highlight recent technological developments enabling epitranscriptomic profiling and discuss future research directions for integrating RNA modification studies into poultry biology and precision livestock production (Figure 1). By leveraging high-throughput strategies such as nanopore direct RNA sequencing and next-generation sequencing, researchers can now overcome previous technical limitations and map these modifications with unprecedented precision (20). Furthermore, these advanced sequencing approaches enable accurate identification of transcript isoforms and complex base-level modifications, which are pivotal for elucidating the molecular mechanisms governing avian physiological development (21).

**Figure 1:**
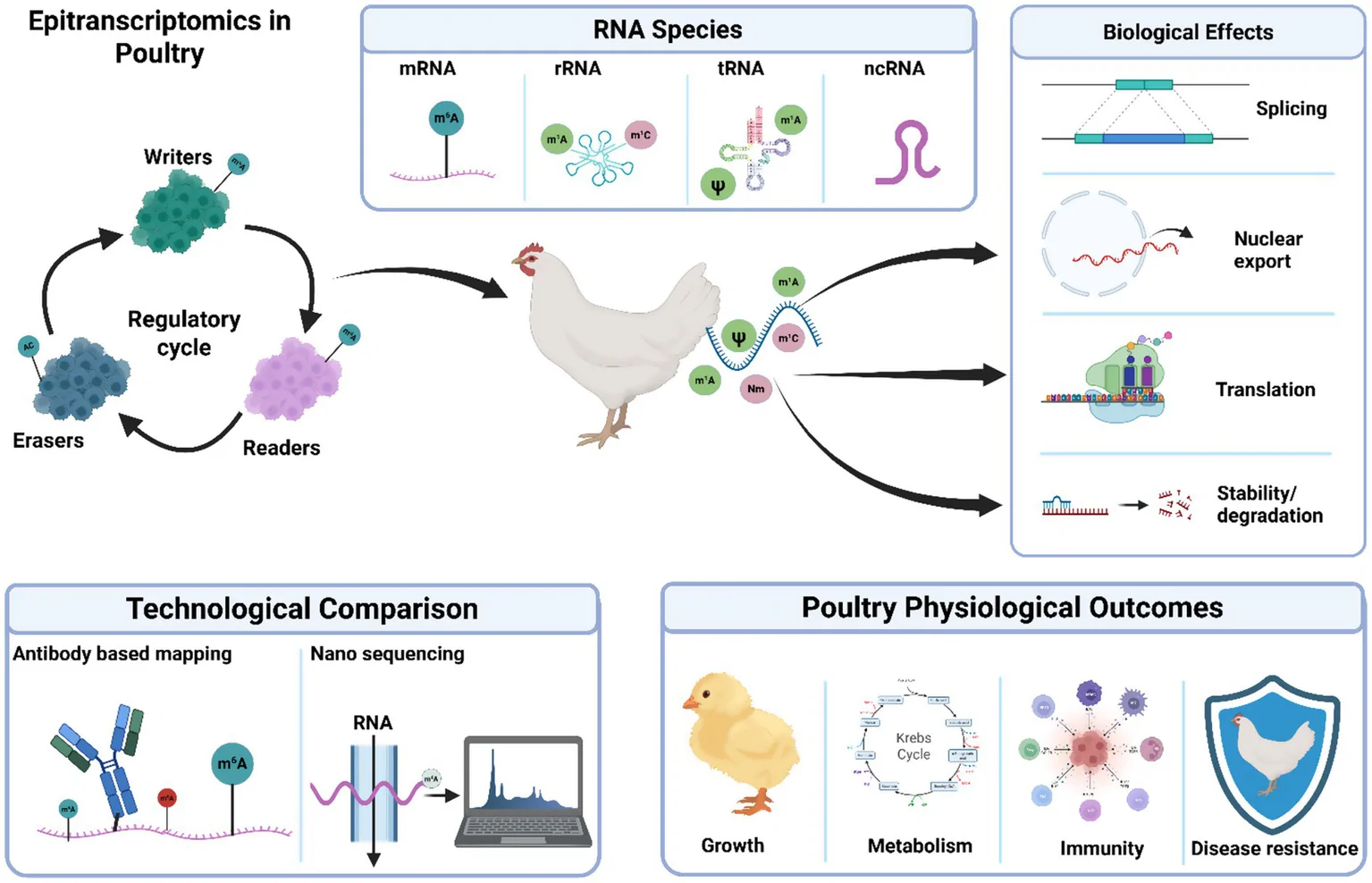
Overview of epitranscriptomic regulation in poultry. Schematic summary of the scope of this review. **(A)** The writer–reader–eraser regulatory cycle: Writer enzymes deposit chemical modifications on RNA, reader proteins bind and interpret the modified RNA, and eraser enzymes remove selected modifications, enabling dynamic and reversible control of gene expression. **(B)** RNA species that carry modifications: Messenger RNA (mRNA; m^6^A, m^5^C, m^1^A), ribosomal RNA (rRNA; m^6^A, m^5^C), transfer RNA (tRNA; m^6^A, Ψ), and non-coding RNA (ncRNA). **(C)** Biological effects of RNA modifications on target transcripts: Splicing, nuclear export, translation, and stability/degradation. **(D)** Comparison of detection technologies: Antibody-based mapping (MeRIP-seq, miCLIP) and nanopore direct RNA sequencing of native RNA. **(E)** Poultry physiological outcomes proposed to be influenced by epitranscriptomic regulation: Growth, metabolism, immunity, and disease resistance. Arrows indicate the direction of the regulatory cycle and its downstream consequences. m^6^A, N^6^-methyladenosine; m^5^C, 5-methylcytosine; m^1^A, N^1^-methyladenosine; Ψ, pseudouridine; Nm, 2′-O-methylation; ncRNA, non-coding RNA. Created with BioRender.com.

### 1.1 Literature search strategy

To improve transparency and reproducibility, relevant literature was identified through structured searches of PubMed, Web of Science, Scopus, and Google Scholar, with the final search completed on 2 May 2026; the search was repeated before manuscript revision to capture newly published poultry epitranscriptomic studies and relevant methodological advances. No restriction was placed on publication year, although priority was given to studies published within the last 10 years.

Searches combined terms for RNA modifications with terms for poultry species and physiological processes. The PubMed search string was: (“epitranscriptomics” OR “RNA modification” OR “m6A” OR “N6-methyladenosine” OR “m5C” OR “5-methylcytosine” OR “m1A” OR “pseudouridine” OR “2’-O-methylation” OR “RNA methylation”) AND (“poultry” OR “chicken” OR “*Gallus gallus*” OR “broiler” OR “layer” OR “duck” OR “avian” OR “goose”) AND (“growth” OR “muscle development” OR “metabolism” OR “nutrition” OR “immunity”). Equivalent keyword combinations were adapted to the search syntax of Web of Science, Scopus, and Google Scholar, and reference lists of relevant articles were additionally examined to identify studies not retrieved through the initial database searches.

Because this article is a narrative review rather than a systematic review, no formal PRISMA screening flow with numerical record counts was performed. Records retrieved from the databases were nonetheless managed systematically: duplicates were removed using reference management software before screening, and titles and abstracts were screened against predefined inclusion criteria: (i) peer-reviewed original research or authoritative reviews addressing RNA modification biology, detection technologies, regulatory machinery, or the roles of RNA modifications in growth, metabolism, or immunity; (ii) studies providing RNA-modification profiles or functional evidence in chickens or other poultry species were given special emphasis; and (iii) because mechanistic evidence in poultry remains limited, mechanistic studies from mammalian and other vertebrate models were included to provide comparative context. Exclusion criteria were: (i) non-peer-reviewed sources, including unreviewed preprints; (ii) studies not available in English; and (iii) studies in which the methodology did not permit reliable detection of RNA modifications, such as enrichment experiments without appropriate controls or validation.

Studies that passed screening were evaluated for relevance to the scope of the review, methodological quality (antibody specificity, validation of modification calls, appropriate controls, and biological replication), and contribution to understanding poultry epitranscriptomics. Evidence obtained directly from chickens or other poultry species was distinguished from comparative evidence derived from non-avian models, and mechanistic claims were weighted accordingly throughout the review.

## 2 The epitranscriptomic landscape

Epitranscriptomics encompasses many chemical modifications in RNA that work together to regulate RNA structure, function, and stability (18). These modifications allow RNA to regulate itself beyond what is encoded in the nucleotide sequence, providing for dynamic and reversible regulation of gene expression. Researchers have identified 170 known types of RNA modifications across multiple species. Therefore, RNA regulation is highly complex. Historically, the study of RNA modifications focused on tRNA and rRNA. However, high-throughput sequencing techniques have now shown widespread modification of mRNA and many other classes of non-coding RNAs (2, 22). These chemical markers serve as a crucial layer of post-transcriptional gene regulation, determining RNA stability and fate and influencing diverse biological phenotypes (23). Of these, $N^{6}$ -methyladenosine ( $m^{6}A$ ) has emerged as the most abundant internal modification in eukaryotic mRNA, acting as a dynamic switch regulated by methyltransferase “writers,” demethylase “erasers,” and specific reader proteins (24, 25). Together with this basic regulatory triad, the coordinated action of these enzymes also controls the establishment, recognition, and removal of chemical tags, leading to outcomes including transcript degradation and translational efficiency changes in translational efficiency (26). More recent studies have specifically implicated these $m^{6}A$ dynamics in modulating avian lipid metabolism and adipogenesis as a potential molecular basis for fat deposition in poultry (27).

In eukaryotes, RNA modifications are not randomly distributed among transcripts but rather tend to cluster in discrete regions, suggesting that they may have regulatory functions. RNA modifications can influence many aspects of RNA metabolism, including splicing, nuclear export, translation efficiency, and RNA turnover. Epitranscriptomic regulation includes chemical modifications of mRNA, tRNA, rRNA, and regulatory non-coding RNAs, such as m^6^A, m^5^C, m^1^A, pseudouridine (Ψ), and 2′-O-methylation (Nm) (1, 28). Evidence in poultry has mainly focused on m^6^A, with studies reported in chicken (*Gallus gallus*) tissues and developmental models, including skeletal muscle and embryonic development. Evidence for m^5^C, m^1^A, Ψ, and Nm is relatively limited. In chicken, changes to RNA-modification profiles have been linked to processes such as muscle development, cellular differentiation, metabolism, and responses to environmental or infectious challenges (3, 29). However, much of the current mechanistic understanding still comes from mammalian systems, and poultry studies often provide transcriptome-wide associations rather than direct functional validation. The poultry epitranscriptomic landscape is therefore an emerging field in which the distribution and functional significance of individual RNA modifications are being gradually unveiled (30).

Non-coding RNAs are also part of the epitranscriptome. RNA modifications can occur not only on mRNA but also on microRNAs (miRNAs), long non-coding RNAs (lncRNAs), circular RNAs (circRNAs) and other regulatory RNA molecules. Modifications of these RNAs can affect their processing, stability, secondary structure, subcellular localization, and interactions with RNA-binding proteins or target RNAs (30, 31). For example, modifying precursor or mature miRNAs may affect their processing and target recognition, while modifying lncRNAs or circRNAs may influence stability and regulatory interactions. These processes link RNA chemistry to post-transcriptional gene regulation and provide an additional level through which epitranscriptomic mechanisms can affect growth, metabolism, and immunity in poultry (32).

### 2.1 Diversity of RNA modifications

Methylation of the nitrogen at the N6 position of adenosine. The methylation of the nitrogen at the N6 position of adenosine gives N6-methyladenosine (m^6^A). An RNA methyltransferase complex transfers the methyl group from S-adenosyl-L-methionine (SAM) to adenosine. m6A in mRNA is frequently enriched near stop codons, in long internal exons, and in 3’ untranslated regions. This modification can change RNA structure and affect its interaction with RNA-binding proteins, which in turn can modulate splicing, nuclear export, transcript stability, translation, and RNA degradation (3, 31, 33).

5-Methylcytosine (m^5^C) is a derivative of cytosine in which a methyl group is added to the carbon-5 position. The RNA m^5^C is mainly catalyzed by NSUN-family methyltransferases and, for selected RNA substrates, by TRDMT1/DNMT2. This modification in several RNA classes, including mRNA, tRNA, and rRNA, where it can affect RNA stability, structural integrity, transport, and translational regulation (34). In addition, other modifications such as pseudouridylation (Ψ) and A-to-I editing are distinct biochemical modifications that contribute to the complexity of the avian epitranscriptome by facilitating important changes in RNA metabolism, structure, and intermolecular interactions (35, 36). Moreover, specific protein complexes read these diverse chemical marks to decipher the epitranscriptomic code and promote complex gene expression programs at different developmental stages (37). This crosstalk between writer complexes and reader proteins is directed by consensus sequences like the RRACH motif, which is often associated with $m^{6}A\;$ (38).

N1-methyladenosine(m^1^A) is generated by methylation at the N1 position of adenosine. Compared to m^6^A, m^1^A has a positive charge under physiological conditions and may affect standard Watson–Crick base pairing. m^1^A is highly prevalent in tRNA and rRNA and is involved in RNA folding, structural stability, and translational fidelity (39, 40). Its presence in mRNA has also been reported, but its distribution and functional significance in mRNA are not as well characterized.

Pseudouridine (Ψ), an isomer of uridine, arises from the enzymatic transformation of the standard carbon–nitrogen glycosidic linkage of uridine to a carbon–carbon bond. Such structural rearrangement can provide an additional hydrogen-bonding capacity and may enhance RNA structural stability. Pseudouridylation is catalyzed either by stand-alone pseudouridine synthases or by H/ACA small nucleolar ribonucleoprotein complexes and is particularly abundant in tRNA and rRNA. However, it is also present in mRNA and other RNA species (25, 41).

2’-O-methylation (Nm) involves the addition of a methyl group to the ribose sugar at the 2′-hydroxyl rather than to the nitrogenous base per se. Fibrillarin-containing C/D box small nucleolar ribonucleoprotein complexes or substrate-specific methyltransferases typically catalyze this modification. Nm can increase resistance to RNA degradation and affect RNA folding, ribosome function and translational accuracy (28, 42).

Collectively, these modifications differ in chemical structure, catalytic machinery, RNA substrates, and functional consequences. The biochemical diversity of RNA molecules provides the molecular basis of the epitranscriptome and confers regulatory properties on RNA beyond its primary nucleotide sequence. Epitranscriptomic marks dynamically alter these physical and chemical parameters of RNA metabolism and act as a regulatory layer that coupe environmental stimuli with cellular homeostatic processes (43, 44). The reversibility of some marks, such as m^1^A, in particular, allows for dynamic control of RNA stability and translation efficiency by methyltransferases and demethylases, including the ALKBH family of proteins (45, 46).

### 2.2 Distribution of RNA modifications across RNA molecules

Many RNA molecules (mRNA, tRNA, rRNA, and noncoding RNAs) undergo a wide variety of post-transcriptional modifications. Among the most extensively studied RNA modifications are N6-methyladenosine (m6A) and 5-methylcytosine (m5C). In mRNA, m6A is commonly enriched near stop codons, within coding regions, and in 3′ untranslated regions, whereas m5C occurs across several RNA classes and can also be detected within functionally relevant regions of mRNA. The distribution influences how these processes occur (e.g., translation initiation, mRNA stability, and interaction with RNA-binding proteins) (1, 3, 37, 47). Notably, 2’-O-methylation is prevalent in both internal coding sequences and the mRNA cap and acts as a critical regulator of gene expression and protein abundance (48). Such modifications are fundamentally linked to ribosomal and transfer RNA biogenesis, where they play pivotal roles in maintaining translational accuracy and structural integrity (49).

tRNA modifications contribute to correct tRNA folding and affect decoding accuracy (i.e., ensuring the right amino acid is placed in the polypeptide chain) (6, 50). As such, rRNA modifications play a vital role in ribosome structure and function, and therefore in protein synthesis efficiency, by modifying the ribosome’s functional components. Regulatory non-coding RNAs, including miRNAs, lncRNAs, and circRNAs, can also carry RNA modifications that influence their maturation, stability, structure, localization, and interactions with target molecules (30, 37). Their modification therefore represents an additional component of epitranscriptomic regulation rather than a separate regulatory process. The fact that modifications exist for all types of RNA supports the idea that RNA modifications are a critical component of cell regulation (1, 16).

### 2.3 Evolutionary conservation of RNA modifications

The evolutionary conservation of RNA modifications indicates their functional importance in biology. Several RNA-modification pathways and homologous regulatory enzymes are evolutionarily conserved across diverse organisms; however, the composition, substrate specificity, regulatory context, and biological roles of these systems can differ substantially among bacteria, fungi, plants, and animals. Core components of the m6A regulatory machinery are conserved across many vertebrate species, including mammals and birds, although their tissue-specific functions and physiological consequences may differ. Therefore, epitranscriptomic regulation is likely an ancient way to adjust gene expression (33, 51).

Genomic and transcriptomic resources are now available for a growing number of bird species, and comparative analyses have confirmed that the core components of the RNA modification machinery, including m6A writers, readers, and erasers, are conserved at the sequence level in chickens and other poultry species (3, 27). However, sequence conservation alone does not demonstrate functional equivalence, and experimentally validated epitranscriptomic mechanisms remain scarce in birds. To date, demonstrated poultry findings are largely limited to transcriptome-wide association studies, such as m6A profiling in chicken skeletal muscle, adipose tissue, and embryonic development, and to a small number of functional studies, for example the role of ALKBH5 in chicken preadipocyte differentiation (24, 52). These studies establish that RNA modifications occur in poultry and are associated with production-relevant processes. Still, they do not yet prove that the conserved machinery regulates avian growth, metabolism, or immunity in the same manner as in mammals. Whether the conserved pathways control physiological traits such as growth efficiency, metabolic adaptation, and immune variation in poultry therefore remains a hypothesis that requires direct experimental testing in avian systems (Figure 2). By leveraging advanced high-throughput sequencing technologies—such as MeRIP-seq—researchers can now begin to profile these epitranscriptomic markers across diverse avian tissues to elucidate their specific roles in post-transcriptional gene regulation (53).

**Figure 2:**
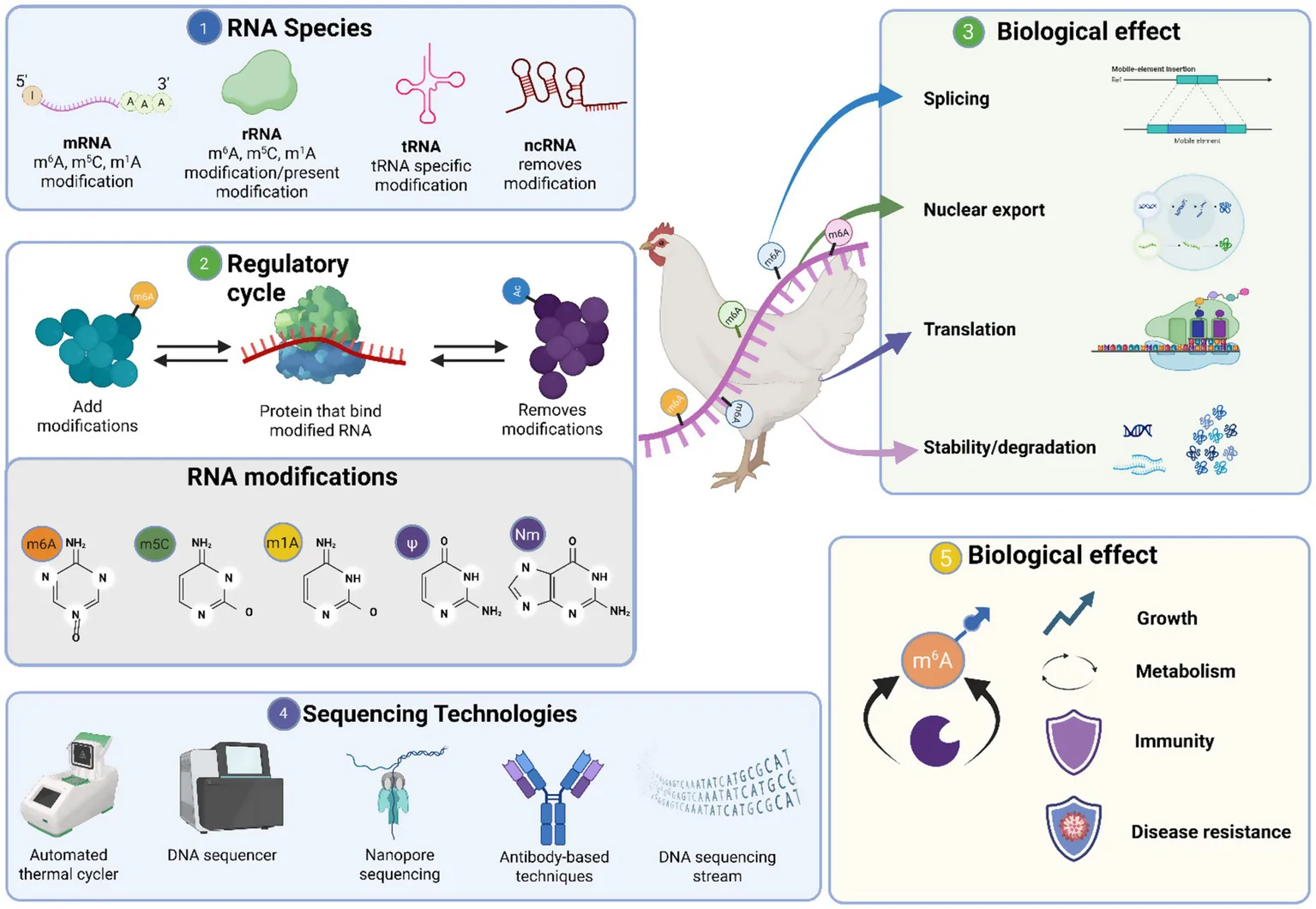
Major RNA modifications and their functional relevance in poultry epitranscriptomics. (1) RNA species that carry modifications: mRNA (m^6^A, m^5^C, m^1^A), rRNA (m^6^A, m^5^C, m^1^A), tRNA (dense, modification-specific patterns), and ncRNA (modifications modulate ncRNA stability and function). (2) The regulatory cycle of RNA modification: Enzymes add modifications (“writers”), proteins bind the modified RNA (“readers”), and enzymes remove modifications (“erasers”; established for m^6^A and selected m^1^A sites only). Chemical structures of the five major modifications (m^6^A, m^5^C, m^1^A, Ψ, Nm) are shown below panel 2. (3) Molecular-level biological effects of modified transcripts: Splicing, nuclear export, translation, and stability/degradation. (4) Sequencing technologies used for modification detection: Automated thermal cycling, DNA sequencing, nanopore direct RNA sequencing, and antibody-based enrichment techniques. (5) Organism-level outcomes proposed to be influenced by RNA modifications: Growth, metabolism, immunity, and disease resistance. Evidence for panels 3 and 5 derives mainly from non-avian systems and remains to be functionally validated in poultry. Created with BioRender.com.

Furthermore, integrating RNA mass spectrometry with high-resolution sequencing enables mapping these modifications under various physiological stressors, providing insight into how avian cells fine-tune gene expression to maintain homeostasis (54). This field of epitranscriptomics, while currently transitioning from basic characterization to functional interrogation, mirrors the complexity seen in the more established field of epigenetics (55). Recent evolutionary studies indicate that the high conservation of the m6A regulatory machinery among vertebrates suggests these modifications are under selective pressure (56), potentially mirroring the functional constraints identified in mammalian epitranscriptomic landscapes (57).

## 3 Molecular machinery of epitranscriptomic regulation

Epitranscriptomic regulation involves modification-specific enzymes and RNA-binding proteins that install, recognize, or, for some modifications, remove chemical marks from RNA. For m^6^A, the major methyltransferase machinery includes the catalytic proteins METTL3 and METTL14 together with regulatory components such as WTAP, VIRMA/KIAA1429, and RBM15/RBM15B. m^6^A-modified transcripts can subsequently be recognized by YTH-domain proteins, including YTHDF1–3 and YTHDC1/2, as well as other modification-responsive RNA-binding proteins. FTO and ALKBH5 can catalyze m^6^A demethylation in defined cellular contexts. Other modifications use distinct machinery: m^5^C is deposited principally by NSUN-family methyltransferases and TRDMT1, whereas pseudouridylation is catalyzed by PUS-family enzymes or H/ACA ribonucleoprotein complexes, and 2′-O-methylation can involve fibrillarin-containing C/D box snoRNP complexes (25, 37, 58).

In chickens, the presence and expression of conserved components of these pathways provide a molecular basis for investigating epitranscriptomic regulation of avian gene expression. Changes in the activity or abundance of these enzymes and RNA-binding proteins could alter RNA stability, splicing, translation, and transcript turnover associated with muscle development, metabolism, and immune responses. However, direct functional characterization of many of these regulators in chickens remains limited; therefore, their proposed physiological effects in poultry should be distinguished from mechanisms established in mammalian systems (59–61).

The writer–reader–eraser framework should not be applied uniformly to all RNA modifications. Although m^6^A is regulated by well-characterized installation, recognition, and removal systems, other modifications differ substantially in their enzymatic machinery, recognition mechanisms, and reversibility (4, 18). For example, m^5^C is deposited principally by NSUN-family methyltransferases and DNMT2/TRDMT1, whereas experimentally established active demethylation mechanisms are less clearly defined than for m^6^A. Pseudouridine is generated by pseudouridine synthases, including PUS-family enzymes, or by guide-RNA-dependent H/ACA ribonucleoprotein complexes, but no broadly accepted enzymatic “eraser” system has been established. Similarly, 2′-O-methylation is catalyzed by site-specific methyltransferases or fibrillarin-containing C/D box snoRNP complexes, whereas general reader and eraser systems remain incompletely characterized. Accordingly, the regulatory machinery of each modification is considered separately below, and the absence of an established reader or eraser should not be interpreted as evidence that such a factor necessarily exists (62, 63). Regarding 5-methylcytosine (m^5^C), current evidence highlights NSUN family proteins and TRDMT1 as the primary methyltransferases (64, 65). At the same time, readers like YBX1 and ALYREF are critical for mediating downstream effects such as enhanced mRNA stability and nuclear export (66, 67).

### 3.1 Writers: RNA modification enzymes

Enzymes called writer proteins add chemical modifications to RNA, shaping the epitranscriptomic landscape. Many RNA modifications exist, and one of the most studied is N6-methyladenosine (m6A). A multi-protein methyltransferase complex places the m6A modification on the RNA molecule; it contains core components that enable methyl transfer (catalytic) activity and recognize which RNA molecule is the substrate (i.e., the RNA to which the methyl is being added). Within the target RNA transcript, the methyltransferases identify specific consensus sequence motifs that are recognized by the methyltransferase enzyme, and these consensus sequence motifs are where the methyltransferases attach (catalyze) a methyl group from S-adenosylmethionine to the specific nucleotide(s) that are targeted (68, 69).

The core m^6^A writer complex contains METTL3, which provides the principal catalytic activity, and METTL14, which contributes to RNA substrate recognition and stabilizes the methyltransferase complex. Additional regulatory components include WTAP, VIRMA/KIAA1429, RBM15/RBM15B, and ZC3H13, which influence localization, substrate selection, and m^6^A deposition on specific transcripts. The methyl donor for this reaction is S-adenosyl-L-methionine (SAM). Distinct enzymes install other RNA modifications; for example, NSUN-family methyltransferases and TRDMT1 catalyze m^5^C, PUS-family enzymes or H/ACA ribonucleoprotein complexes generate pseudouridine, and fibrillarin-containing C/D box snoRNP complexes catalyze 2′-O-methylation (1, 37, 70). Similarly, the N1-methyladenosine (m1A) landscape is orchestrated by a separate suite of methyltransferases, including TRMT61A, TRMT6, TRMT10C, and TRMT61B, which ensure the precise installation of these modifications on tRNA, rRNA, and mRNA species (71).

The alteration in m6A mainly develops during the processes of transcription and in the advancement of an mRNA molecule through the nucleus. Within mRNA molecules, modified m6As are commonly found in proximity to stop codons, in long internal sections of exons, and at the ends of the untranslated regions of an mRNA strand (the 3’ end). The proximity of these modifications to mRNA suggests they may contribute significantly to mRNA stability and translation levels (3, 33).

Along with m6A methyltransferases, many other writers locally install alternative RNA chemical modifications. For instance, cytosine methyltransferases catalyze the formation of m5C modifications on RNA molecules; other enzymes generate pseudouridine and 2’-O-methylated nucleotides. These modifications are particularly abundant in tRNA and rRNA, where they play key roles in proper RNA folding and structural stability. Together, these writer enzymes establish the chemical diversity of RNA molecules and lay the groundwork for downstream epitranscriptomic regulation (72, 73).

### 3.2 Readers: RNA modification recognition proteins

Enzymes that write RNA modifications deposit them on RNA molecules. Proteins that read these RNA modifications interpret them as chemical marks and translate those marks into functional biological outcomes using RNA-binding domains. Reader proteins use their RNA-binding domains to distinguish between different types of modified nucleotides in an RNA transcript. Reader proteins then bind those modified nucleotides, recruiting other regulatory proteins that influence RNA processing, translation, or degradation (26, 74).

Several RNA-binding protein families recognize m^6^A-modified transcripts and translate the modification into distinct post-transcriptional outcomes. The best-characterized direct m^6^A readers are YTH-domain proteins. Cytoplasmic YTHDF proteins, including YTHDF1, YTHDF2, and YTHDF3, participate in the regulation of translation efficiency and transcript turnover, whereas YTHDC1 functions predominantly in the nucleus and contributes to pre-mRNA processing, alternative splicing, and nuclear export. YTHDC2 has roles in RNA metabolism and translation in selected cellular contexts. Other proteins, including IGF2BP1, IGF2BP2, and IGF2BP3, can preferentially associate with m^6^A-containing transcripts and promote RNA stability and translation in defined contexts. hnRNP-family proteins may also respond indirectly to m^6^A-induced changes in RNA secondary structure, a mechanism often described as an “m^6^A switch” (75–77).

In mRNA splicing, m^6^A can influence the accessibility or recruitment of splicing regulators. Nuclear YTHDC1 can interact with splicing factors such as SRSF3 and modulate exon inclusion and transcript processing. Therefore, the functional consequence of an m^6^A mark depends not only on the presence of the modification but also on which RNA-binding protein recognizes the modified transcript, its cellular location, and the surrounding RNA sequence and structure (78, 79). Furthermore, in certain contexts, these m^6^A-modified transcripts can be sequestered into specialized cytoplasmic compartments, such as stress granules or processing bodies, to regulate their availability for translation or to facilitate targeted mRNA decay (80).

Reader proteins can also help coordinate how RNA molecules and intracellular machinery interact, such as how machinery makes protein or breaks down RNA. For example, some reader proteins enhance translation by recruiting ribosomes to modified RNA transcripts, while others cooperate with decay machinery to promote transcript degradation. Thus, RNA modifications can have many different regulatory effects across different aspects of gene expression networks (81).

Most research on the characteristics of reader proteins has used mammals as models. However, researchers have also identified an abundance of RNA-binding proteins in birds. Further studies are needed to determine how these proteins are involved in epitranscriptomic regulation within avian tissues, including muscle, liver, and immune organs (82). Specifically, the hnRNP family—comprising HNRNPA2B1, HNRNPC, and HNRNPG—warrants investigation in poultry to clarify whether they operate as direct m^6^A readers or, analogous to mammalian models, function through structural m^6^A-switches to modulate avian pre-mRNA splicing and miRNA biogenesis (83–85) (Table 1).

**Table 1:** Modification-specific RNA regulatory machinery, recognition mechanisms, and evidence for reversibility.

RNA modification	Principal installation machinery	Established/putative recognition proteins	Removal/eraser evidence	Reversibility status	References
m^6^A	METTL3–METTL14 catalytic complex with WTAP, VIRMA/KIAA1429, RBM15/15B and other cofactors	YTHDF1–3, YTHDC1/2; IGF2BP proteins and selected heterogeneous nuclear ribonucleoproteins can mediate modification-dependent effects	FTO and ALKBH5 demethylases	Strong evidence for enzymatic reversibility	(4, 92)
m^5^C	NSUN-family methyltransferases; TRDMT1/DNMT2 for selected RNAs	ALYREF and YBX1 reported as m^5^C-binding/recognition factors in defined contexts	No universally accepted dedicated m^5^C RNA eraser comparable with ALKBH5/FTO	Active reversibility remains incompletely established	(26, 92)
m^1^A	TRMT6–TRMT61A, TRMT10-family and other substrate-specific enzymes	Modification-dependent recognition has been reported, but a general canonical reader system is not established	ALKBH1 and ALKBH3 can demethylate selected m^1^A substrates	Reversible for particular substrates	(3, 107)
Ψ	PUS-family pseudouridine synthases and H/ACA snoRNP-guided machinery including dyskerin/DKC1	No universal dedicated Ψ-reader class established	No established general Ψ eraser	Generally considered stable; enzymatic reversal not established	(4, 92)
Nm (2′-O-methylation)	Fibrillarin-containing C/D box snoRNP complexes and substrate-specific methyltransferases	No universal dedicated reader system established	No established general Nm eraser	Enzymatic reversibility not established	(107)

Enzymatic removal has been demonstrated for only a subset of RNA modifications and should not be assumed to be a universal property of the epitranscriptome. For m^6^A, the Fe(II)/α-ketoglutarate-dependent dioxygenases FTO and ALKBH5 can catalyze demethylation in defined cellular contexts. ALKBH-family enzymes can also remove selected m^1^A modifications from particular RNA substrates. In contrast, general enzymatic eraser systems have not been established for pseudouridine or 2′-O-methylation, while active reversal of RNA m^5^C remains substantially less well defined (65, 86).

Where enzymatic reversal has been experimentally demonstrated, changes in modification status may influence transcript stability, translation, or RNA processing. In poultry, the physiological roles of these demethylation pathways remain insufficiently characterized. These pathways allow reversible methylation to be regulated in response to the cellular environment. A key example is m6A demethylation, where m6A modification can be reversed (47, 83).

Eraser enzymes catalyze the oxidative demethylation of modified nucleotides, removing methyl groups and returning the modified base to its original form, thereby erasing the epitranscriptome. Through this reversible mechanism, cells can dynamically adjust RNA methylation patterns in response to environmental, metabolic, or developmental signals (18, 87).

The ability to dynamically add and remove RNA modifications may provide a flexible cellular regulatory scheme that complements classical transcriptional and epigenetic control mechanisms. These eraser enzymes modulate RNA modifications, which influence transcript stability, translation efficiency, and gene expression patterns. Although RNA demethylases have been identified in different vertebrate species, little is known about their functional roles in poultry physiology (88, 89).

### 3.3 Dynamic regulation of RNA modifications

The abundance, localization, and activity of RNA-modifying enzymes and modification-responsive RNA-binding proteins can vary with developmental stage, nutrient availability, cellular metabolism, hypoxia, oxidative stress, and environmental challenge. These responses are modification-specific, and not every RNA modification possesses defined writer, reader, and eraser components. Cellular metabolites such as S-adenosyl-L-methionine and α-ketoglutarate may influence the activity of selected methyltransferases and demethylases, providing a potential connection between metabolic state and RNA-modification dynamics.

Epitranscriptomic mechanisms adjust how cells produce proteins when adapting to physiological changes by affecting the modifications made to an RNA molecule after it has been synthesized (epitranscriptomic). These epitranscriptomic changes help cellular proteins adapt to ever-changing physiological stressors, including high temperatures, exposure to reactive oxidative species, and infectious organisms such as viruses and bacteria (90, 91).

Environmental issues like heat stress, changing nutrition, and infections put birds at risk in poultry production systems. All three can ultimately affect gene regulation in birds’ tissues, metabolism, immune response, and growth. To breed birds that can handle stress and resist disease, a clear understanding of how RNA modifications respond to environmental stressors will be beneficial (9, 92).

Collectively, modification-specific enzymes and RNA-binding proteins provide a dynamic layer of post-transcriptional regulation. Further poultry-specific studies are required to determine how these pathways contribute to physiological adaptation. Further investigations to elucidate these molecular mechanisms will be critical to understanding how this process contributes to the physiological regulation of poultry species through modification of their respective RNAs (52) (Figure 3).

**Figure 3:**
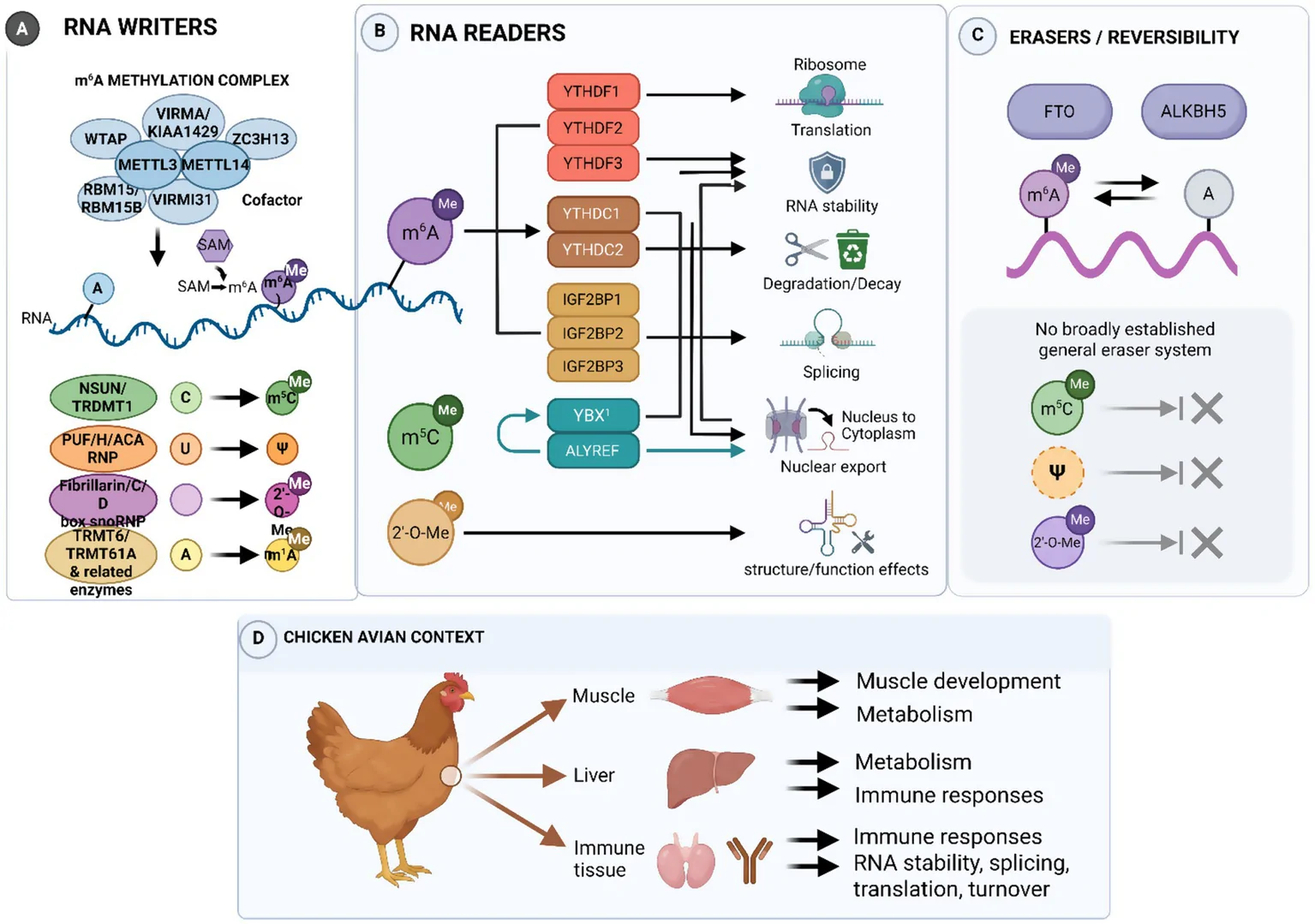
Molecular machinery of epitranscriptomic regulation and its proposed roles in chickens. **(A)** RNA writers: The m^6^A methyltransferase complex METTL3 (catalytic subunit) and METTL14 with accessory proteins WTAP, VIRMA/KIAA1429, ZC3H13, and RBM15/RBM15B transfers a methyl group from S-adenosyl-L-methionine (SAM) to adenosine within RRACH consensus contexts. Other writers install distinct marks: NSUN-family enzymes and TRDMT1 deposit m^5^C; PUS-family pseudouridine synthases and H/ACA snoRNPs generate Ψ; fibrillarin-containing C/D box snoRNPs catalyze 2′-O-methylation (Nm); and TRMT6/TRMT61A and related enzymes deposit m^1^A. **(B)** RNA readers: Modification-responsive RNA-binding proteins interpret the marks. YTHDF1–3 regulate translation, RNA stability, and degradation/decay of m^6^A-modified transcripts; YTHDC1/2 contribute to splicing and nuclear export; IGF2BP1–3 stabilize modified transcripts; m^5^C is recognized by YBX1 and ALYREF, which promote transcript stability and nuclear export, respectively; 2′-O-methylation confers structural and functional effects on RNA. **(C)** Erasers and reversibility: FTO and ALKBH5 catalyze oxidative demethylation of m^6^A, making this mark dynamic and reversible. No broadly established general eraser system exists for m^5^C, Ψ, or Nm, which are therefore considered comparatively stable. **(D)** Chicken avian context: The pathways depicted in **(A–C)** are proposed to operate in chicken muscle (muscle development and metabolism), liver (metabolism and innate immune responses), and immune tissues (immune responses and regulation of RNA stability, splicing, translation, and turnover). Direct functional evidence in poultry remains limited, and most depicted mechanisms require validation in avian systems. m^6^A, N^6^-methyladenosine; m^5^C, 5-methylcytosine; m^1^A, N^1^-methyladenosine; Ψ, pseudouridine; Nm (2′-O-Me), 2′-O-methylation; SAM, S-adenosyl-L-methionine; snoRNP, small nucleolar ribonucleoprotein; RRACH, m^6^A consensus motif (R = A/G; H = A/C/U). Created with BioRender.com.

## 4 Technologies for detecting RNA modifications

Advances in epitranscriptomics have been spurred by new, high-resolution technologies for identifying RNA modifications. Using classic methods of molecular biology for detecting RNA modifications had been quite limited in terms of providing biochemical insight (due to low sensitivity and inability to distinguish single nucleotide modifications), but in the last 10 years, we have seen the advent of very powerful molecular (biochemical); immunological and sequencing technologies that provide transcriptome-wide detection of RNA modifications, and provide the ability to identify and map thousands of RNA modification sites in multiple RNA species with the capability to detect the dynamic nature of epitranscriptomics and how RNA modifications affect, regulate, and control normal physiological functions, such as growth, metabolism, and immunity (18, 20).

In the poultry industry, these technologies are still in early development; however, they could change how poultry and animal scientists study and understand chickens and other avian species at the genome level. Transcriptome-wide profiling of RNA modifications in chickens and other birds will provide insight into the regulatory mechanisms underlying key poultry phenotypes, such as growth efficiency, nutrient utilization, and immune competence (93, 94).

### 4.1 Antibody-based sequencing methods

Immunoprecipitation-based approaches were among the earliest high-throughput techniques designed to map RNA modifications across the entire transcriptome. These profiling methods rely on enriching RNA fragments containing specific chemical modifications using modification-specific antibodies. Next, researchers sequence the RNA fragments using short-read sequencing methods, which allow identification of fragments containing the specific chemical modification of interest (36, 95).

A common immunoprecipitation-based approach for RNA modification profiling is N6-methyladenosine (m6A) RNA immunoprecipitation sequencing (MeRIP-seq). This method uses antibodies that specifically target m6A to capture m6A-containing RNA, which can then be sequenced and aligned to a reference genome to identify enriched regions containing m6A. While MeRIP-seq provides transcriptome-wide m6A profiles, the associated sequencing data typically have a resolution of 100–200 nucleotides. Importantly, MeRIP-seq identifies regions of relative m6A enrichment rather than demonstrating that a specific modification causes a downstream biological phenotype (96, 97). Differentially enriched peaks can be associated with changes in gene expression or pathways, but such associations require independent validation before a causal regulatory mechanism can be inferred. Functional validation may include site-specific modification assays, manipulation of writer or eraser enzymes, reporter assays, loss- or gain-of-function experiments, or rescue studies, which limit the ability to identify exact m6A modification sites (53, 98).

To address this limitation, researchers developed m6A individual-nucleotide resolution crosslinking and immunoprecipitation (miCLIP). In this method, the anti-m6A antibody crosslinks to m6A-modified RNA, which is then used to generate crosslinked RNA for sequencing. Mutations or truncation patterns are detected during reverse transcription, indicating the location of the m6A-modified nucleotide and enabling miCLIP data generation with single-nucleotide resolution. miCLIP has been widely used in mammalian systems to create high-resolution m6A maps, but its application has only recently begun in poultry tissues (99, 100).

A similar approach can be used to profile the other two most commonly studied modifications (5-methylcytosine (m5C) and pseudouridine), as several similar immunoprecipitation-based enrichment strategies are available. In general, antibody-based methods are useful for transcriptome-wide RNA modification mapping; however, they also have limitations. These limitations include variability in antibody modification specificity, the potential for a single antibody to recognize modifications in addition to its intended target, and the inability to detect multiple modifications in a single experiment (101, 102).

### 4.2 Chemical and enzymatic detection methods

Two common options for determining the presence of RNA modifications involve using either chemical or enzymatic techniques. Both techniques exploit differences in the reactivity of unmodified and modified nucleotides (i.e., they produce a specific reaction that either changes the nucleotide or labels the modified form with a marker) to generate a signal detectable by sequencing (103, 104). For example, bisulfite sequencing is commonly used to detect 5-methylcytosines (m5C). When sodium bisulfite is added to RNA, it converts unmethylated cytosines to uracil, whereas m5C remains unchanged. Therefore, when researchers perform bisulfite sequencing on the modified RNA, they can distinguish methylated (m5C) from unmethylated cytosines at single-base resolution. Although this method has been widely used in studies of mammalian RNA, it must be optimized for poultry RNA because mammals and poultry differ substantially in RNA composition and transcriptome complexity (105, 106).

Another frequently used method to detect RNA modifications is mapping pseudouridine (Ψ) positions in RNA (commonly referred to as Ψ-seq). Ψ-seq involves the use of CMC (N-cyclohexyl-N′/−(2-morpholinoethyl) carbodiimide metho-p-toluenesulfonate) or other chemical modification agents that will chemically alter or modify the structure of the pseudouridine within the modified RNA. When this occurs, the modified pseudouridine inhibits reverse transcription less. It thus leads to characteristic stops or mutations in sequence reads, allowing researchers to locate the position of the modified pseudouridine within the RNA transcript (20).

In addition to chemical methods, researchers have also established various enzymatic methods to detect specific RNA modifications. Several enzymes specifically bind to, cleave, or label different types of modified nucleotides and can enrich modified RNA or detect an individual RNA modification at specific points in the transcript. Overall, these methods are quite precise, but their use is typically limited because they allow researchers to identify only one specific modification using one particular enzyme (20, 107).

In general, chemical and enzymatic methods to detect RNA modifications provide higher resolution than antibody-enriched detection methods. However, the requirement for many different, complex experimental procedures is often a limitation to detecting RNA modifications using chemical or enzymatic methods, as it is for immunoprecipitation-based detection of RNA modifications, and similarly limits the utility of these methods for detecting more than one RNA modification simultaneously from the same sequencing run (108, 109).

### 4.3 Long-read sequencing technologies

New sequencing technologies, called “third-generation sequencing” technologies, have greatly expanded the ability to study RNA modifications across the entire transcriptome. Unlike the original ‘short-read’ sequencing techniques, which depended on reverse transcription and amplification to generate cDNA from RNA and then sequence the cDNA, “long-read” sequencing technologies allow researchers to directly analyze native, unaltered, full-length RNA molecules, preserving the modification-related signals that may have been obliterated or lost during the cDNA synthesis process (109, 110).

Nanopore direct RNA sequencing (DRS) has become an important approach for studying epitranscriptomic modifications because it enables analysis of native RNA molecules without obligatory reverse transcription or PCR amplification. As RNA passes through the nanopore, nucleotide-dependent changes in ionic current are recorded, and chemical modifications can alter these signal patterns. This allows modification-associated signals to be examined in the context of full-length transcripts and can support transcript- and isoform-resolved analyses when appropriate computational models and experimental controls are used. However, the accuracy and positional resolution of modification detection depend on several factors, including modification type, sequencing chemistry, read coverage, model training, and the availability of suitable control datasets. Consequently, reliable discrimination of multiple modification types within the same transcript is not universally achievable, and site-level modification assignments often require orthogonal validation using independent biochemical or sequencing-based approaches (111, 112).

Using nanopore DRS to characterize epitranscriptomic modifications in the domestic poultry research community will provide a powerful means to study the RNA modification landscape across diverse tissues, such as liver, spleen, muscle, and immune organs in poultry. Characterizing the epitranscriptomic landscape across poultry tissues will provide information on tissue-specific regulatory mechanisms and how RNA modifications contribute to physiological adaptation, immune responses, and responses to environmental stressors in avian species (27, 47).

A second long-read platform, Pacific Biosciences (PacBio) single-molecule real-time sequencing, is primarily a tool for full-length transcript and isoform characterization rather than a dedicated RNA-modification detection method. PacBio sequencing generates high-fidelity consensus cDNA sequences (HiFi reads), and because it requires reverse transcription, it does not directly read native RNA modifications as in nanopore direct RNA sequencing. Modification information can be recovered only indirectly, for example from reverse transcriptase misincorporation or pausing signatures at modified nucleotides, and such approaches have been explored mainly in specialized assays rather than in routine transcriptome profiling (113, 114). For poultry research, the principal value of PacBio sequencing lies in its accurate, full-length isoform characterization, which complements nanopore direct RNA sequencing datasets and supports transcript-resolved interpretation of modification signals (19, 105). Combining the two platforms therefore strengthens isoform-level analysis of complex avian transcriptomes, whereas modification calling itself should rely on nanopore direct RNA sequencing with appropriate controls.

### 4.4 Computational tools and bioinformatic pipelines

Advanced bioinformatics pipelines for RNA modification detection and interpretation will enable analysis of large-scale sequencing datasets to determine which signals derive from mutations rather than random variation or artifacts. Each modification must also be accurately identified from the temporal signal produced by nanopore sequencing (3rd generation) to determine which nucleotides are modified (20, 36).

Nanopore sequencing datasets use custom software programs, such as Tombo, Nanocompore, and EpiNano, to analyze raw ionic current signal data and provide evidence for the presence of RNA modifications. These software programs can also compare modifications across multiple tissue types, developmental stages, and conditions, revealing dynamic and context-dependent differences in epitranscriptomic patterns (115, 116).

Integrating epitranscriptomic analyses with traditional transcriptomic datasets will enhance functional insights into epitranscriptomic data. Mapping RNA modifications alongside gene expression profiles allows exploration of potential correlations between RNA modifications and transcript stability, alternative splicing, and translational efficiency, helping elucidate how epitranscriptomic mechanisms contribute to gene expression control (16, 117).

Visualizing and annotating modification maps will improve how researchers use epitranscriptomic data to interpret modification landscapes. Visualization platforms and genome browsers can provide an effective way for researchers to visualize RNA modifications across the transcriptome, particularly in the 5’ UTR, CDS, and 3’ UTR, facilitating the identification of potential regulatory hotspots (3, 36).

Combining long-read sequencing technologies with established bioinformatics frameworks will synergistically enhance the research experience and produce a comprehensive epitranscriptomic profile. Applying these methodologies in poultry research will enable characterization of tissue-specific RNA modifications that influence growth, metabolism, and immunity, and will provide a biological basis for precision nutrition and the development of advanced breeding techniques (27, 118).

### 4.5 Poultry-relevant RNA-modification databases and reusable datasets

Public RNA-modification databases provide important resources for re-analysis, cross-study comparison, and prioritization of candidate modification sites; however, poultry representation remains substantially lower than in human and mouse datasets. m^6^A-Atlas v2.0 contains m^6^A data across 42 species and includes *Gallus gallus*, although chicken coverage is predominantly based on lower-resolution enrichment data and remains limited compared with major mammalian models. RMBase v3.0 provides broader coverage, integrating 73 RNA-modification types across 62 species, including *G. gallus*, and enables exploration of modification sites, associated genes, motifs, and regulatory annotations (119–121). RMDisease v2.0 also includes chicken and provides information on genetic variants potentially associated with the gain or loss of RNA-modification sites. MODOMICS complements these resources by providing curated information on RNA-modification chemistry, modified residues, enzymes, and biosynthetic pathways, although it is not primarily a poultry-specific transcriptomic atlas (122, 123). Consider these specialized resources alongside primary repositories such as NCBI GEO and SRA, where raw and processed datasets from individual poultry epitranscriptomic studies may be available for independent re-analysis. Existing poultry datasets include transcriptome-wide m^6^A and m^5^C profiling from chicken skeletal muscle, embryonic muscle development, and infection-associated tissues, together with m^6^A datasets from other poultry species. Nevertheless, avian datasets remain fragmented across tissues, developmental stages, breeds, experimental conditions, and analytical platforms. A standardized poultry epitranscriptomic resource that integrates dataset accession numbers, species, tissue, modification type, detection method, genome assembly, and evidence resolution would substantially improve data reuse, reproducibility, and comparative analyses (36, 117) (Table 2).

**Table 2:** Major RNA-modification databases and their relevance to poultry epitranscriptomic research.

Database	Scope	Chicken/poultry content	Evidence type for chicken sites	Primary use in poultry research
m6A-Atlas v2.0	m6A modification across 442 species	*Gallus gallus* included	Predominantly lower-resolution enrichment data; limited coverage compared with mammalian models	Cross-study comparison and candidate m6A site prioritization
RMBase v3.0	73 RNA-modification types across 62 species	*Gallus gallus* included	Mixture of experimentally supported and computationally predicted sites; support level must be checked per record	Exploration of modification sites, associated genes, motifs, and regulatory annotations
RMDisease v2.0	Genetic variants associated with gain or loss of RNA-modification sites	Chicken included	Predicted variant-modification associations	Prioritization of variants in poultry disease and trait studies
MODOMICS	RNA-modification chemistry, modified residues, enzymes, and biosynthetic pathways	Not species-specific	Curated biochemical reference data	Annotation of modification chemistry and machinery in poultry transcripts
NCBI GEO/SRA	Raw and processed datasets from individual studies	Growing but fragmented poultry datasets (m6A and m5C profiles from chicken skeletal muscle, embryonic muscle development, infection-associated tissues, and other poultry species)	Experimentally derived; platform- and study-dependent	Independent re-analysis, integration across studies, and reuse of primary poultry epitranscriptomic data

## 5 Role of RNA modifications in poultry growth and development

Bird growth is a complex trait that relies on coordinated gene expression across multiple tissues, including muscle, liver, and the gastrointestinal tract. Beyond these forms of gene expression, epitranscriptomic mechanisms add another layer of regulation that can fine-tune the transcriptional program through RNA-based mechanisms, modulating protein production, metabolic activity, and cellular differentiation. Recent studies in mammals have shown that RNA modifications such as 6 N-methyladenosine (m6A), 5 N-methylcytosine (m5C), and pseudouridine (Ψ) can influence stem cell development, organ formation, and growth factor receptor signaling. Although few direct studies exist in poultry, the conservation of epitranscriptomic processes suggests these mechanisms also play similar regulatory roles in birds (27, 92, 124).

### 5.1 m6A modifications and muscle development

Evidence in this section is drawn mainly from mammalian myogenesis studies; where poultry studies exist, they are identified as such. In mammals, m6A marks mRNA encoding the myogenic transcription factors MyoD and Myogenin, regulating both transcript stability and translation efficiency and thereby influencing the rate of myoblast differentiation (125–127). In poultry, IGF-1 is an established regulator of muscle growth, but direct evidence that m6A modification of IGF-1 transcripts controls chicken muscle accretion remains insufficient. Transcriptome-wide m6A profiling in broiler skeletal muscle has identified modification patterns associated with divergent muscle growth between hypertrophic and leaner lines (128), Still, such profiles are associative, and causal involvement of individual m6A sites will require targeted functional validation (128, 129).

### 5.2 tRNA modifications and protein synthesis

Several important tRNA modifications, including N1-methyladenosine (m1A) and 2′-O-methylation (Nm), contribute to translational fidelity, RNA stability, and efficient protein synthesis. These functions are particularly relevant in rapidly growing tissues with high protein-synthesis demands, such as skeletal muscle. Studies in mammalian systems show that disrupting specific tRNA modifications can reduce translational efficiency, impair protein synthesis, and alter amino acid metabolism. These findings suggest that tRNA modification may also influence growth-related processes in poultry; however, direct evidence linking specific tRNA modifications to growth performance or feed efficiency in chickens remains limited and requires targeted experimental validation (130, 131).

### 5.3 RNA modifications in nutrient-responsive growth regulation

RNA modifications, such as m6A and m5C, are known to impact the length of time mRNAs are active (i.e., their half-life) or the amount of protein produced from an mRNA. For example, if there are enough nutrients, the m6A modification will increase translation of the protein that helps the organism grow through its 5’ UTR; however, if there are not enough nutrients (i.e., limited nutrient availability), the addition of m6A may be reversed to conserve energy for the down-regulation of mRNA translation (132).

In chickens, additional mechanisms regulate feed efficiency and growth under different nutritional regimens. Therefore, epitranscriptomic modifications provide potential targets for developing innovative precision nutrition. Identifying tissue-specific epitranscriptomic modifications in liver and muscle will provide information on transcripts regulated by epitranscriptomic modifications in response to dietary intake (133, 134).

### 5.4 Non-coding RNAs and growth regulation

Long non-coding RNAs (lncRNAs) and microRNAs (miRNAs) both play significant roles in regulatory pathways controlling growth-related genes. Studies have found that certain RNA modifications in non-coding RNAs can influence their stability and maturation, and therefore how they interact with the mRNAs they target. Modification by m6A of non-coding RNAs, for example, can create alterations in the secondary structure of lncRNA and directly affect whether the lncRNA can recruit chromatin-modifying complexes or act as a miRNA sponge (3, 37).

RNA modifications of non-coding RNAs may represent an additional regulatory layer affecting myogenesis, nutrient sensing, and endocrine signaling in poultry. However, most mechanistic evidence linking modification-dependent regulation of lncRNAs or miRNAs to these processes has come from mammalian systems. In poultry, these relationships should therefore be considered testable hypotheses rather than established mechanisms until direct modification mapping and functional perturbation studies are available (27, 135).

In poultry, epitranscriptomic regulation of non-coding RNA will likely play a key role in refining the transcriptional networks associated with myogenesis, nutrient sensing, and endocrine signaling. This additional layer of regulation may account for the observed differences in growth traits among breeds and individuals reared in different environments (59).

### 5.5 Environmental and hormonal influence on RNA modifications

Factors such as temperature, stocking density, and feed may affect poultry growth performance, along with other non-genetic factors. Emerging evidence indicates that different types of environmental stress affect RNA modification levels. For example, heat stress can alter m6A RNA modification profiles on messenger RNAs involved in protein synthesis and/or stress response. By modifying messenger RNA stability and/or translation, epitranscriptomic modifications can also integrate hormonal signals from the endocrine system and transduce them to cells for optimal growth (16, 23).

## 6 Epitranscriptomic control of metabolism in poultry

Poultry’s growth, productivity, and health are dependent on metabolic regulation. The liver, muscles, and adipose tissues work together to regulate energy intake, nutrient use, and synthesis. Although the role of transcriptional and post-translational regulation in metabolism has long been recognized, the role of epitranscriptomic modifications in fine-tuning +/− regulation of gene expression in response to external and nutritional influences remains to be established (55, 136).

RNA modifications such as m6A, m5C, pseudouridine, and Nm regulate transcripts encoding metabolic enzymes, nutrient transporters, and signaling proteins in mammalian systems. Direct evidence for equivalent mechanisms in poultry remains comparatively limited. Conservation of core epitranscriptomic machinery supports the biological plausibility of similar regulatory functions in avian species, but conservation alone does not demonstrate functional equivalence. Accordingly, we use mammalian findings as comparative mechanistic evidence and distinguish them from experimentally demonstrated poultry-specific observations (93, 137).

### 6.1 m6A-mediated regulation of metabolic enzymes

Several metabolic transcripts, including those encoding enzymes involved in glycolysis, gluconeogenesis, fatty-acid synthesis, and amino-acid metabolism, have been associated with m6A-dependent regulation in mammalian models. In poultry, genes such as PK, G6Pase, ACC, and FAS should therefore be regarded as candidate targets for epitranscriptomic investigation rather than confirmed m6A-regulated metabolic effectors. Transcriptome-wide enrichment or pathway analyses can prioritize such candidates, but causal regulation requires direct site-level and functional validation (138, 139).

### 6.2 tRNA and ribosomal RNA modifications in translation efficiency

The functions described in this paragraph are established mainly in mammalian and microbial systems; direct evidence in poultry is lacking. In mammals, tRNA modifications such as m1A, m5C, and Nm contribute to translational fidelity, RNA stability, and efficient protein synthesis, and disruption of specific tRNA modifications reduces translational efficiency and alters amino acid metabolism (130, 131, 140). Such functional groups in tRNA, N1-methyladenosine (m1A), 5-methylcytosine (m5C), and 2′-O-methylation (Nm), and pseudouridine and Nm within rRNA modify the structural stability and functional optimization of this translation system (37, 141).

These modifications maintain precise codon-anticodon pairing during translation, ensuring effective decoding of mRNA encoding highly expressed metabolic enzymes. They also provide structural integrity and stability to ribosomes, supporting the high rates of protein synthesis needed to sustain rapid tissue growth and metabolic activity. In addition, dynamic changes in tRNA and rRNA modification patterns can modulate translation under nutrient limitation or metabolic stress, allowing selective synthesis of proteins essential for metabolic adaptation (140, 142).

Disruptions or alterations in any of these RNA modification patterns will adversely affect translation efficiency, impair protein synthesis capacity, and perturb metabolic pathways. Accordingly, the available comparative evidence suggests that proper epitranscriptomic regulation of tRNA and rRNA represents a biologically plausible, but not yet poultry-validated, mechanism that may contribute to the metabolic homeostasis required for optimal growth performance in poultry; this proposed relationship requires direct experimental confirmation in avian systems (142, 143).

### 6.3 RNA modifications in nutrient-sensing and metabolic signaling

The relationships described below have been demonstrated in mammalian and other non-avian models and should be regarded as comparative evidence. Whether m6A or m5C regulate components of the mTOR, AMPK, or insulin signaling pathways in poultry has not been directly tested. Evidence from mammalian and other non-avian models indicates that RNA modifications, particularly m^6^A and m^5^C, may influence the expression, stability, or translation of transcripts associated with these pathways. However, comparable modification-dependent mechanisms have not yet been comprehensively established in poultry. Therefore, components of the mTOR, AMPK, and insulin-signaling pathways should currently be considered candidate targets of epitranscriptomic regulation in avian species rather than confirmed downstream effectors (144, 145).

Changes in cellular nutrient and energy status can alter RNA-modifying enzyme activity and thereby influence post-transcriptional regulation in defined experimental systems. For example, m^6^A-related regulatory mechanisms have been linked in non-avian models with transcripts involved in mTOR-associated growth signaling and AMPK-regulated metabolic adaptation. At the same time, m^6^A- and m^5^C-associated regulation has also been reported for components of insulin-responsive pathways. These observations provide a mechanistic framework for investigating whether similar relationships occur in poultry. Still, they should not be interpreted as direct evidence that RNA modifications regulate these pathways in chickens. Tissue-specific modification mapping, combined with expression analysis and functional perturbation of modifying enzymes or candidate RNA sites, will be required to establish causality (146–148).

RNA modifications may also modulate insulin signaling, a pathway vital for regulating glucose uptake and storage. In mammalian models, m6A and m5C marks on transcripts encoding the insulin receptor (INSR) or its downstream effectors can modulate transcript stability or translation, thereby influencing glucose metabolism and glycogen storage (149, 150).

If these mechanisms are conserved in birds, RNA modifications could provide an additional layer of regulatory control, potentially enabling the reprogramming of metabolic processes in poultry in response to changes in diet composition, feeding schedules, and energetic demands and supporting efficient nutrient utilization and growth. Because these mechanisms have been demonstrated primarily in non-avian models, this proposed role in poultry remains a hypothesis that requires direct testing in avian systems (118) (Figure 4).

**Figure 4:**
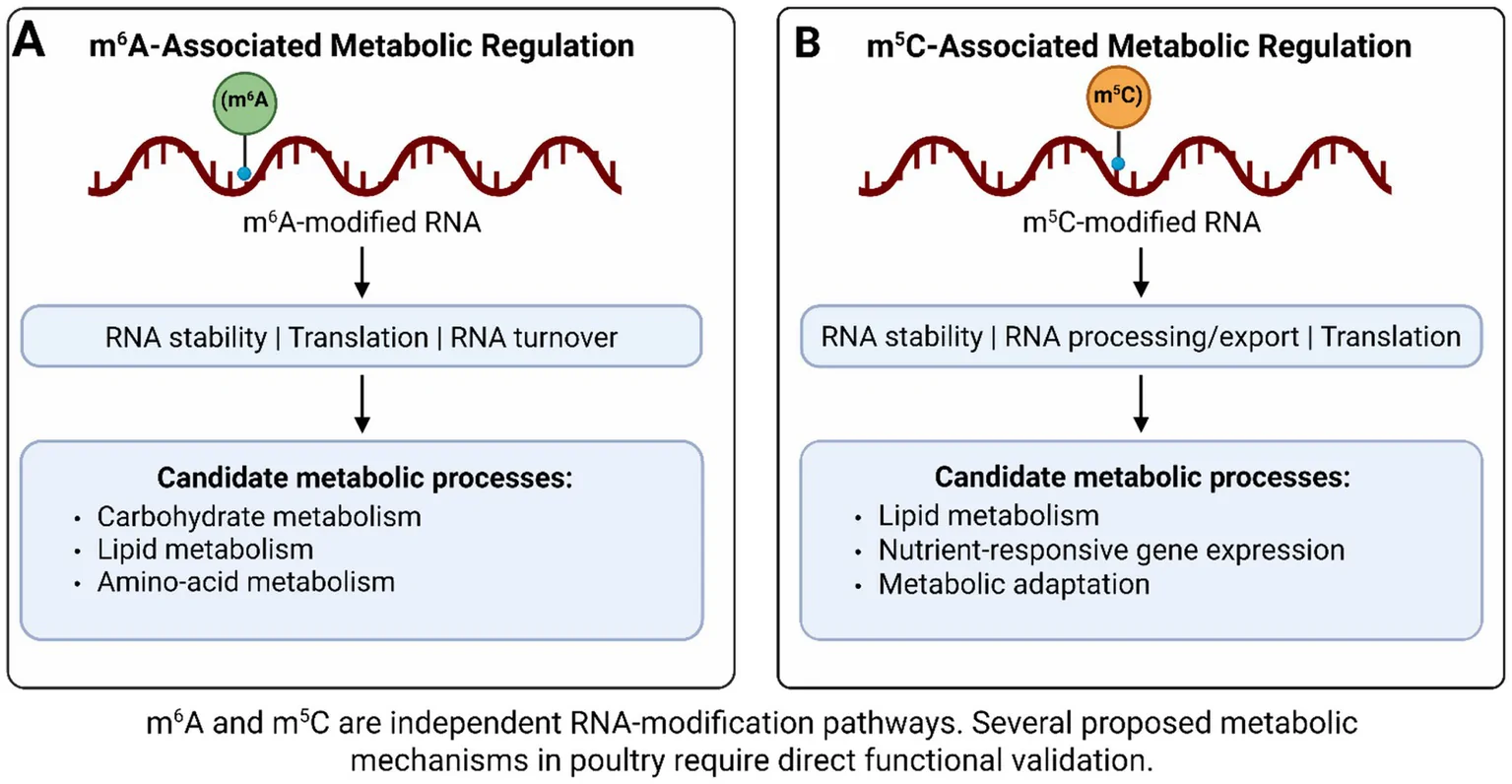
Proposed roles of m^6^A and m^5^C in metabolic regulation. **(A)** M^6^A-associated metabolic regulation: M^6^A-modified RNAs undergo reader-dependent changes in RNA stability, translation, and turnover, which have been associated in mainly non-avian studies with candidate metabolic processes including carbohydrate metabolism, lipid metabolism, and amino-acid metabolism. **(B)** M^5^C-associated metabolic regulation: M^5^C-modified RNAs show altered stability, processing/nuclear export, and translation, which have been associated with candidate metabolic processes including lipid metabolism, nutrient-responsive gene expression, and metabolic adaptation. M^6^A and m^5^C are independent RNA-modification pathways with distinct writer and reader machineries. Outcomes shown in dashed boxes denote reported associations and candidate mechanisms derived largely from non-avian models; their causal roles in poultry metabolism require direct functional validation in avian systems. M^6^A, N^6^-methyladenosine; m^5^C, 5-methylcytosine. Created with BioRender.com.

### 6.4 Non-coding RNA modifications in metabolic control

lncRNAs and miRNAs are major players involved in controlling metabolic gene expression. RNA modifications also regulate the function of non-coding RNAs. Epitranscriptomic RNA modifications affect multiple functional aspects of non-coding RNAs, such as transcript stabilization, processing, and interactions with target molecules; thereby, they add a layer of regulation to metabolic processes (12, 151).

Modifications to lncRNAs can alter their stability and degradation, thereby affecting their role as molecular scaffolds or regulators of metabolic gene expression. Similarly, modifications to primary (or precursor-derived) miRNAs can alter miRNA maturation by changing processing via the Drosha and Dicer complexes, thereby changing the total abundance of mature miRNAs. These changes can alter miRNA target recognition and the post-transcriptional regulation of genes encoding metabolic enzymes, nutrient transporters, and regulatory proteins (152, 153).

Through these mechanisms, RNA modification coordinates the interplay between metabolic and growth-related signals and links cellular nutrient status to transcriptional and post-transcriptional regulatory networks. Identifying modified lncRNAs and miRNAs, particularly in metabolically active tissues (e.g., liver or skeletal muscle) in poultry, may reveal previously unrecognized regulatory pathways that influence feed usage, fat storage, and adaptation to metabolic stress (154, 155).

## 7 Epitranscriptomics in poultry immunity

Precise gene expression regulation in the avian immune system is key to mounting an effective immune response against pathogens while maintaining overall homeostasis within host tissues. One way the avian immune system achieves this precise control over gene expression is through epitranscriptomic modification (i.e., alteration of RNA products after transcription). Many recent studies conducted in mammals have identified RNA modifications as critical components of innate and adaptive immunity, including m6A, m5C, pseudouridine (Ψ), and 2′-O-methylation (Nm).

Although understanding of how these RNA modifications affect the immune system in poultry is limited, the conservation of RNA modification machinery across vertebrates suggests that similar regulatory effects may be observed in chickens and other avian species (124, 156).

Most mechanistic knowledge regarding epitranscriptomic control of innate and adaptive immunity originates from mammalian systems, whereas direct functional evidence in poultry remains comparatively sparse. Accordingly, the immune mechanisms discussed below are classified according to the strength and origin of the available evidence. Findings demonstrated directly in chickens or other poultry species are identified as poultry-specific evidence. In contrast, mechanistic observations derived from mammalian or other non-avian systems are presented as comparative evidence. Proposed roles of RNA modifications in avian immune tissues, pathogen responses, and vaccine responsiveness are therefore treated as hypotheses requiring poultry-specific experimental validation rather than as established causal mechanisms.

### 7.1 m6A modifications in innate immunity

Innate immunity is the body’s first line of defense against foreign pathogens. This defense relies on the rapid activation (via transcription) of immune-related products such as cytokines, chemokines, and ISGs (interferon-stimulated genes). Growing evidence also supports the importance of RNA modifications, specifically methylation, in potently modulating innate immune responses. More specifically, RNA modifications (e.g., m6A) may play critical roles in regulating the degradation, translation, and turnover of immune transcripts (157, 158). For instance, m6A methylation critically regulates immune cell proliferation, differentiation, and overall functional capacity, ensuring rapid responses to external threats (8).

In mammalian systems, m6A modifications regulate immune cell proliferation, differentiation, and functional capacity (157, 158). In poultry, direct functional evidence is limited. Still, transcriptome-wide studies have profiled m6A dynamics in chicken liver following avian leukosis virus subgroup J infection and in duckling liver during attenuated duck hepatitis A virus infection, and direct RNA sequencing has revealed chicken post-transcriptional modification responses to *Campylobacter jejuni* inoculation (9). These studies show that pathogen exposure alters RNA modification landscapes in poultry, although the causal contribution of individual marks to immune outcomes remains largely untested (159, 160). Beyond influencing transcript stability, m6A RNA modifications may also promote the translational efficiency of certain immune transcripts. During infection, m6A-mediated regulation of immune transcript translation may enable rapid synthesis of specific antiviral and antimicrobial proteins needed for host cells to mount an adequate defense against invading pathogens (161, 162). RNA modifications may also influence innate immune signaling by regulating transcripts encoding PRRs (pattern recognition receptors) (e.g., TLRs). m6A RNA modification may modulate PRR transcript levels, thereby affecting PRR expression and the sensitivity and magnitude of downstream innate immune signaling pathways (163).

In poultry, variegated epitranscriptomic regulation will likely occur in many of the important immune system tissues, including the spleen, bursa of Fabricius, and circulating immune cells. Transcriptome-wide mapping of RNA modification landscapes from these infectious disease-relevant immune system tissues, using long-read sequencing technologies such as nanopore direct RNA sequencing, should provide insight into the overall epitranscriptome landscape of various immune system tissues and how RNA modification affects the immune response to the pathogen and the responsiveness of poultry to vaccination (18).

### 7.2 tRNA and rRNA modifications in immune cell function

For immunological protection, immune cells that need to produce cytokines, chemokines, and surface receptors quickly require efficient cellular processes to make those proteins. Modifications to transfer RNA (tRNA) (i.e., N1-methyladenosine (m1A), 5-methylcytosine (m5C), and 2′-O-methylation (Nm)) and ribosomal RNA (rRNA) (i.e., pseudouridine (Ψ) and Nm) contribute to translational accuracy and ribosomal stability (4, 37).

In turn, these modifications play critical roles in rapid, accurate protein synthesis in immune cells by facilitating cytokine production in macrophages and dendritic cells, antibody synthesis in B cells, and T-cell population expansion and differentiation. Disruptions or alterations in tRNA or rRNA modification patterns can impair translational efficiency and may reduce immune response effectiveness (164).

Studying tRNA and rRNA modification dynamics in poultry immune organs could clarify cellular mechanisms that regulate protein synthesis during immune activation and help develop methods to enhance disease resistance and improve vaccine effectiveness in birds (18, 165).

### 7.3 Non-coding RNA modifications in immune regulation

Non-coding RNAs, such as long non-coding RNAs (lncRNAs) and microRNAs (miRNAs), regulate the immune response through epitranscriptomic modifications. Various chemical modifications (e.g., N6-methyladenosine (m6A) or Pseudouridine (Ψ)) on lncRNAs are known to increase stability and help regulate the production of cytokines and other immune mediators (166, 167).

Likewise, epitranscriptomic modifications to miRNAs influence miRNA maturation and subsequent interaction with their target genes, resulting in fine-tuned post-transcriptional control of immune gene expression. Modified non-coding RNAs can also regulate their own production or activity by acting as molecular sponges or decoys, helping regulate the balance between pro-inflammatory and anti-inflammatory signaling and ultimately preventing over-activation of the immune system (168, 169).

In chickens, epitranscriptomic regulation of lncRNAs and miRNAs may direct the host response against viral pathogens such as avian influenza virus (AIV) and Newcastle disease virus (NDV), as well as bacterial infections; ultimately, this may improve overall flock health, disease resistance, and vaccine response (170, 171).

### 7.4 Dynamic regulation during infection and stress

RNA modifications in immune tissues may be dynamically influenced by environmental stressors such as heat, overcrowding, or nutrient deficiency and can, in turn, affect immune responses. For example, heat stress has been shown to alter m6A deposition on transcripts encoding cytokines in mammalian models; whether poultry immune tissues show the same response has not been directly demonstrated. Likewise, nutrient restriction may alter RNA modification patterns in immune tissues to shift energy toward critical defense processes. Pathogen exposure has also been shown to cause global changes in RNA modification patterns; as a result, genes involved in the expression of virus-suppressing or bacteria-suppressing genes may be increased to enhance the immune system’s ability to protect against pathogen s (8).

Dynamic changes in epitranscriptomics provide an additional regulatory mechanism that allows poultry to adapt their immune system to changes in environmental and pathogen-related conditions; thus, this dynamic regulatory mechanism supports poultry resilience and overall flock health (172) (Figure 5).

**Figure 5:**
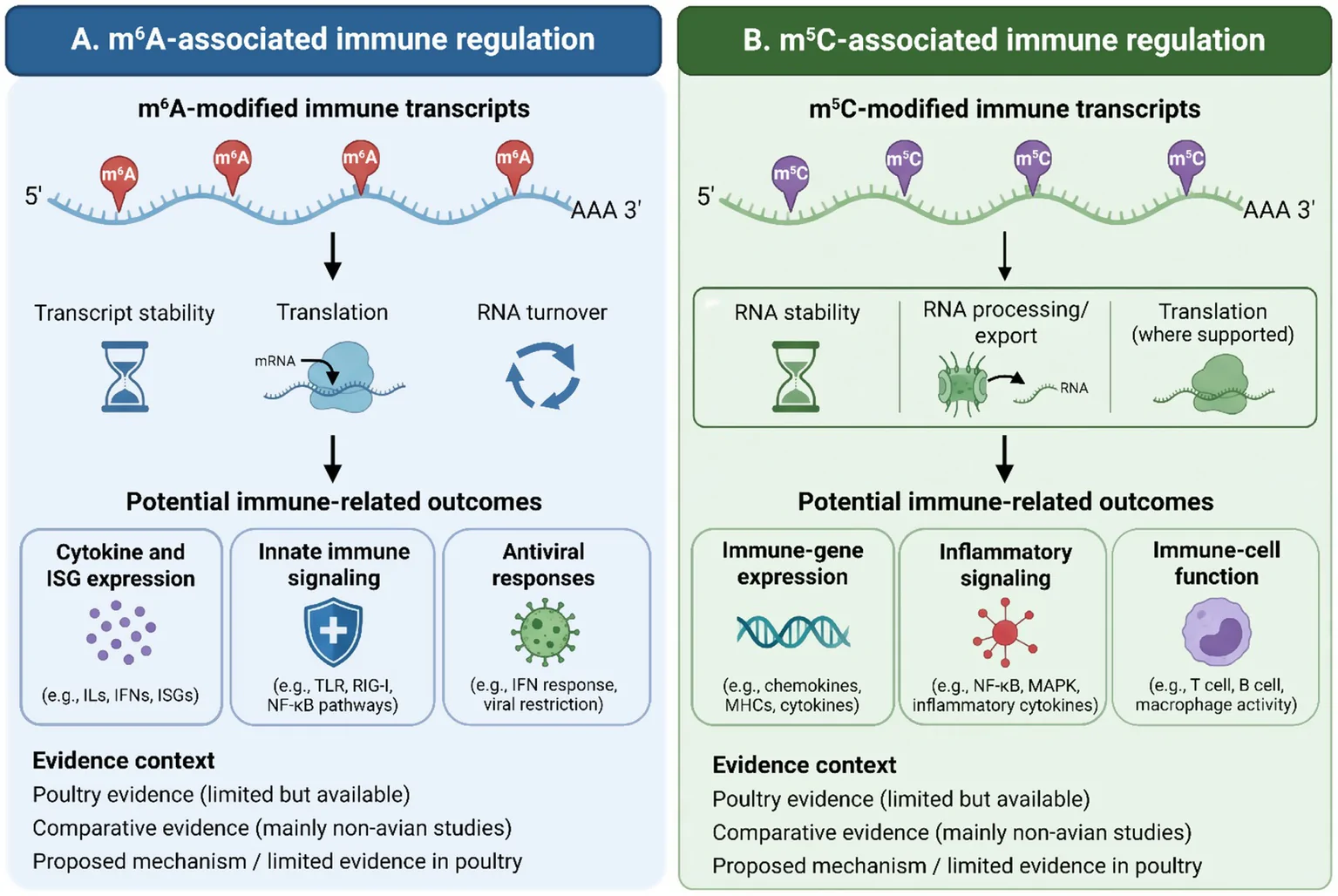
Proposed roles of m^6^A and m^5^C in immune regulation. **(A)** m^6^A-associated immune regulation: m^6^A-modified immune transcripts undergo reader-dependent changes in transcript stability, translation, and turnover, with potential outcomes including cytokine and interferon-stimulated gene (ISG) expression (e.g., interleukins, IFNs, ISGs), innate immune signaling (e.g., TLR, RIG-I, and NF-κB pathways), and antiviral responses (e.g., IFN response and viral restriction). **(B)** m^5^C-associated immune regulation: m^5^C-modified immune transcripts show altered stability, processing/nuclear export, and where supported translation, with potential outcomes including immune-gene expression (e.g., chemokines, MHC molecules, cytokines), inflammatory signaling (e.g., NF-κB and MAPK pathways), and immune-cell function (e.g., T-cell, B-cell, and macrophage activity). Classify the evidence base of each outcome: Direct poultry evidence (limited but available); comparative evidence derived mainly from non-avian studies, proposed mechanism with limited evidence in poultry. Direct poultry evidence includes transcriptome-wide m^6^A profiling in chicken liver following avian leukosis virus subgroup J (ALV-J) infection and in duckling liver during duck hepatitis A virus infection; most depicted mechanisms remain to be functionally validated in avian systems. m^6^A, N^6^-methyladenosine; m^5^C, 5-methylcytosine; ISG, interferon-stimulated gene; IFN, interferon; IL, interleukin; TLR, Toll-like receptor; MHC, major histocompatibility complex; NF-κB, nuclear factor kappa B; MAPK, mitogen-activated protein kinase. Created with BioRender.com.

## 8 Multi-omics integration: linking epitranscriptomics to poultry physiology

When examining RNA modifications functionally, it is important to consider other molecular layers of information, such as gene expression, protein abundance, and metabolic activity. Multi-omics analyses allow geneticists to link epitranscriptomic changes to how chickens perform in terms of growth rate, feed-conversion efficiency, immune competence, or ability to withstand adverse conditions (173).

### 8.1 Integration with transcriptomics

RNA sequencing (RNA-seq) tells researchers what is expressed; epitranscriptomics provides insight into how chemical modifications reveal the stability, translation, and activity of that transcript. Integrating both datasets helps researchers understand the role of RNA modifications in functional outcomes at the transcriptomic level (174).

For instance, the alteration of mRNA transcript levels (the number of mRNA molecules present at a point in time) and/or splice patterns (the pattern in which the mRNA has been modified) can be linked to the deposition of the epitranscriptomic modifications of m6A or m5C, thereby enabling researchers to determine how modified RNA influences gene expression. Furthermore, specific epitranscriptomic profiles in a given tissue or in response to specific environmental conditions may associate with gene networks that regulate growth, metabolism, and immune functions. As comparative evidence from non-avian systems, transcriptome-wide m6A profiling of porcine muscle has shown that mRNAs with higher levels of m6A are associated with more efficient translation, thereby contributing to protein accumulation during rapid growth; whether the same association holds in poultry muscle remains to be determined (175, 176).

This integration has already been applied in poultry. For example, paired long-read and short-read RNA sequencing of chicken tissues has resolved transcriptome complexity comparable to that of human and mouse, providing isoform-resolved frameworks onto which modification data can be layered (105, 177). Transcriptome-wide m6A profiling combined with RNA-seq in broiler skeletal muscle has linked modification patterns to differences in muscle growth between hypertrophic and leaner lines, and m6A profiling of chicken liver during ALV-J infection has connected modification dynamics to immune-related transcript changes (156). In these studies, transcriptomic integration was correlational; functional confirmation of individual modification sites remains to be performed (7, 118, 156).

### 8.2 Integration with proteomics

Proteomic analysis measures protein abundance and post-translational modifications. Proteomic analysis also gives us a functional view of gene expression. RNA modifications add another layer of regulation by changing mRNA stability, translation efficiency, and thus the amount of protein produced from a given mRNA (178). In poultry, combined transcriptomic and proteomic analyses have been applied to post-hatch hepatic development in chickens, revealing metabolic reprogramming that single-omic approaches missed (179). Such studies demonstrate the feasibility of multi-omic integration in avian tissues, although none has yet incorporated epitranscriptomic data; adding a modification layer to existing poultry transcriptome-proteome datasets, for instance from muscle development studies, would be a logical next step (60). By integrating proteomic and epitranscriptomic data, researchers can identify RNA modifications that correspond to increased or decreased levels of specific proteins, associate certain modifications with enzyme activities involved in metabolism, muscle development, or protein production for the immune system, and resolve discrepancies between mRNA and protein levels through the effects of RNA modification-based regulation (3).

A multi-omics approach to chicken studies provides a unique opportunity to better understand biological processes that control chicken growth, nutrient use efficiency, and metabolic regulation. This information can ultimately help develop strategies to improve chicken productivity and health (179, 180).

### 8.3 Integration with metabolomics

Concrete poultry examples come from transcriptomic-metabolomic studies of hepatic lipid metabolism in laying hens and from analyses of microRNA regulation of chicken hepatic lipid metabolism, which jointly identified regulatory nodes controlling fat deposition (106, 154). These studies show that multi-omic integration can resolve metabolic regulation in poultry, but they did not include modification-level data. Incorporating m6A or m5C profiles into such designs would test whether RNA modifications explain residual variance between transcript abundance and metabolite flux. Metabolomics provides a snapshot of cellular biochemical networks by measuring small molecules and metabolites produced by cells, reflecting the state of metabolic pathways. Furthermore, by regulating the expression or translation of an enzyme or nutrient transporter, RNA modifications can indirectly alter the abundance and flow of metabolic molecules (25).

Using both metabolomics and epitranscriptomics allows identification of new modified transcripts linked to variations in metabolite profiles within metabolically active tissues such as muscle and liver. This approach is powerful for understanding how RNA modifications dynamically regulate nutrient-sensing pathways and energy metabolism, and it can help identify biomarkers for improved feed efficiency, animal performance, and adaptive responses to environmental or nutritional stressors in poultry (181).

### 8.4 Systems-level insights and applications

Combining transcriptomics, proteomics, metabolomics, and epitranscriptomics creates a comprehensive picture of each system’s role in poultry physiology. In particular, by overlaying RNA modification patterns in specific tissues with the structure, function, and metabolism of the proteins in those same tissues, we will better understand how RNA modifications affect the growth, metabolism, and immunity of poultry animals (27, 182).

Demonstrated examples of systems-level integration in poultry include combined genomic and transcriptomic analyses of heat stress response and multi-tissue full-length transcriptome resources in chickens, both of which provide foundations for adding epitranscriptomic layers. Proposed applications that remain to be tested include using epitranscriptomic biomarkers for precision breeding, designing nutritional interventions that modulate methyl-donor availability to influence RNA modification pathways, and optimizing vaccination strategies through modification profiling of immune genes. Comparative mammalian evidence supports these translational directions, but avian systems have not yet validated them (177, 183).

## 9 Future perspectives

Epitranscriptomics is rapidly emerging as a transformative frontier in poultry biology, offering insights into how RNA modifications dynamically regulate gene expression in response to developmental, nutritional, and environmental cues. Unlike static genomic information, RNA modifications provide a flexible, reversible layer of control, allowing tissues such as liver, muscle, and immune organs to fine-tune growth, metabolism, and defense mechanisms in real time. With the advent of high-throughput long-read sequencing technologies, particularly nanopore direct RNA sequencing, researchers can now capture full-length transcriptomes along with multiple modification types, opening new avenues to explore tissue-specific and isoform-specific epitranscriptomic landscapes in chickens and other avian species (105, 177).

Integrating epitranscriptomic profiling with complementary multi-omics datasets promises a systems-level understanding of poultry physiology. By linking RNA modifications to transcript abundance, protein levels, and metabolite flux, researchers can uncover the regulatory networks that coordinate growth, feed efficiency, immune competence, and stress resilience. Emerging computational tools, including machine learning-based pipelines for modification detection, will further enhance the precision and resolution of these analyses, enabling the identification of subtle but biologically meaningful patterns. At the same time, functional genomics approaches, such as CRISPR-mediated perturbation of writer, reader, and eraser proteins, can validate causal relationships between specific modifications and physiological outcomes, bridging the gap between correlation and mechanism (184, 185).

From an applied perspective, epitranscriptomics holds immense potential for poultry production. RNA modifications could serve as biomarkers for precision breeding, allowing the selection of birds with superior growth, metabolism, or disease resistance. Nutritional interventions designed to optimize methyl-donor availability or other cofactors may modulate RNA modification pathways, enhancing protein synthesis, metabolic efficiency, and immune responses. Similarly, understanding the epitranscriptomic regulation of immune genes could improve vaccination strategies and pathogen resilience, contributing to healthier and more productive flocks. By linking molecular insights to practical management strategies, RNA modification research can drive sustainable gains in productivity, health, and welfare (25, 186).

Despite these exciting opportunities, several challenges remain. Poultry-specific tissue and condition datasets remain limited, and the complexity of RNA modifications demands standardized experimental and computational pipelines for robust detection and interpretation. The high costs and technical expertise required for long-read sequencing and integrated multi-omics analyses can also be barriers, especially for large-scale population studies. Addressing these limitations through collaborative research, shared resources, and methodological innovations will be crucial to realizing the full potential of epitranscriptomics in poultry science (52, 187).

Looking ahead, the field is poised for rapid expansion. Advances in sequencing technology, bioinformatics, and functional validation will likely reveal novel RNA modifications and regulatory mechanisms that underpin growth, metabolism, immunity, and stress adaptation. Integrating these insights with practical breeding, nutritional, and health strategies promises to revolutionize poultry production, offering a molecular roadmap for precision agriculture that balances productivity, animal welfare, and sustainability (188).

## 10 Conclusion

Epitranscriptomics represents an emerging regulatory frontier in poultry biology, offering a dynamic layer of gene regulation that complements genomic, epigenomic, and transcriptomic control mechanisms. As synthesized in this review, RNA modifications including m^6^A, m^5^C, m^1^A, pseudouridine (Ψ), and 2′-O-methylation regulate important aspects of RNA metabolism, including transcript stability, splicing, nuclear export, translational efficiency, and RNA degradation. These functions provide a strong biological basis for investigating their potential roles in poultry growth and development, metabolic homeostasis, immune function, and adaptation to environmental stress. However, the strength of evidence supporting specific physiological effects in poultry varies considerably among modifications and biological processes. The convergence of technological innovations, particularly nanopore direct RNA sequencing and integrated multi-omics approaches, has substantially expanded the capacity to profile RNA modification landscapes. Combined with transcriptomic, proteomic, and metabolomic datasets, epitranscriptomic profiling can help identify regulatory networks associated with growth, nutrient utilization, immune responses, and stress resilience in poultry. These approaches may provide valuable molecular insights relevant to future breeding, nutritional, and health-management strategies.

Nevertheless, interpret these potential applications cautiously until modification-associated phenotypes and regulatory pathways are functionally validated in avian systems. Despite these opportunities, several important challenges remain. Poultry-specific datasets are still limited, and much of the available mechanistic knowledge has been extrapolated from mammalian or other non-avian models. Accordingly, interpret the current evidence according to its experimental origin and strength. Distinguish direct findings from chickens or other poultry species from mechanistic evidence from mammalian or non-avian systems, and treat proposed avian regulatory relationships as hypotheses requiring experimental validation. In particular, associations identified through MeRIP-seq, differential RNA-modification profiling, transcriptomic correlations, or pathway-enrichment analyses can identify candidate regulatory relationships. Still, they should not be interpreted as demonstrated causal mechanisms. Functional validation studies that perturb specific RNA-modifying enzymes, RNA-binding proteins, or modification sites in avian systems are therefore urgently needed to establish causal relationships between RNA modifications and physiological outcomes. Site-specific validation, loss- and gain-of-function approaches, rescue experiments where appropriate, and phenotype-level analyses will help strengthen mechanistic conclusions. Standardized experimental protocols and robust bioinformatic pipelines are also essential to improve reproducibility and interpretation across studies. Collaborative research networks, shared data repositories, and methodological advances will be critical for overcoming these limitations. Looking ahead, continued advances in sequencing chemistry, computational analysis, and functional genomics are expected to improve understanding of epitranscriptomic regulation in poultry. Establishing the biological significance of specific RNA modifications in avian systems may ultimately provide new opportunities to support sustainable, efficient, and health-resilient poultry production while balancing productivity, animal welfare, and environmental sustainability.
